# Predictive Factors for Early-Onset Seizures in Patients With Cerebral Venous Sinus Thrombosis

**DOI:** 10.3389/fneur.2022.842807

**Published:** 2022-03-29

**Authors:** Shuwen Mu, Jun Li, Kunzhe Lin, Yi Fang, Feng Lin, Ziqi Li, Yongjun Xu, Shousen Wang

**Affiliations:** ^1^Department of Neurosurgery, Fuzong Clinical Medical College of Fujian Medical University, Fuzhou, China; ^2^Department of Neurosurgery, 900th Hospital, Fuzong Clinical Medical College of Fujian Medical University, Fuzhou, China; ^3^Department of Neurosurgery, Affiliated Fuzhou First Hospital of Fujian Medical University, Fuzhou, China; ^4^School of Medicine, Xiamen University, Xiamen, China; ^5^Laboratory of Basic Medicine, 900th Hospital, Fuzong Clinical Medical College of Fujian Medical University, Fuzhou, China

**Keywords:** cerebral venous sinus thrombosis, seizure, superior sagittal sinus, cerebral hemorrhage, NIHSS, D-dimer

## Abstract

Seizures are reported to be important factors contributing to poor prognosis in patients with cerebral venous sinus thrombosis (CVST). However, the predictive factors for concurrent early onset seizures in patients with CVST remain unclear. To identify the predictive factors of early seizures in patients with CVST, this study retrospectively evaluated the clinical data of patients diagnosed with CVST at two centers from January 2011 to December 2020 and analyzed the relationship between admission characteristics and early onset seizures. A total of 112 CVST patients (63 men and 49 women; mean age 39.82 ± 15.70 years) were enrolled in this study, of whom 34 (30.36%) had seizures. For patients with seizures, cerebral hemorrhage, cortical vein thrombosis, anterior superior sagittal sinus (SSS) thrombosis, middle SSS thrombosis, CVST score, modified Rankin Scale, National Institute of Health Stroke Scale (NIHSS) score, neutrophil percentage, and D-dimer level were more severe than those without seizures. Logistic regression analysis showed that cerebral hemorrhage (*P* = 0.002), anterior SSS thrombosis (*P* = 0.003), NIHSS score ≥5 (*P* = 0.003), and D-dimer ≥0.88 mg/L (*P* = 0.004) were all significant predictive factors of early-onset seizures in CVST patients. Combining the four factors further improved the predictive capability with an area under the curve of 0.871 (95% confidence interval = 0.803–0.939). Further large-scale prospective studies are required to confirm these findings.

## Introduction

Cerebral venous sinus thrombosis (CVST) is a predisposing factor for stroke, with an annual incidence of ~3–4 cases per million people (1). CVST usually occurs in younger adults compared to other types of stroke (2). The clinical manifestations of CVST lack specificity, ranging from isolated headaches to local neurological symptoms, seizures, and coma (3). The prognosis for most patients is favorable. Previous studies have shown that more than half of patients achieve good functional outcomes (4).

However, studies have found that CVST complicated by seizures is an unfavorable prognostic factor, and CVST patients with seizures have a threefold higher mortality rate than those without seizures (5). Seizures can be divided into two types according to the different periods during which they occur during the course of CVST: early onset seizures (occurring before diagnosis or within the first 2 weeks after diagnosis) and late seizures (more than 2 weeks after diagnosis) (6). A previous study reported a high incidence of seizures in CVST patients (42.77%) (7). The mechanisms underlying early onset seizures remain unclear. Although some studies have attempted to identify potential predictive factors for seizures, such as motor or sensory deficits, cerebral hemorrhage, and cortical vein thrombosis (8–11), there is still no clear consensus regarding the predictive value of these risk factors. Therefore, it remains of clinical importance to study predictive factors for early onset seizures in patients with CVST with the aim of making an early diagnosis and providing timely treatment to potentially vulnerable patients.

## Methods

### Study Population

This study retrospectively analyzed the clinical data of patients diagnosed with CVST at the Fuzong Clinical Medical College and Affiliated Fuzhou First Hospital of Fujian Medical University between January 2011 and December 2020. Acute and subacute patients were selected from the database and 112 patients with CVST were included.

The inclusion criteria were as follows: (1) age >18 years and (2) diagnosis of CVST based on magnetic resonance imaging (MRI), magnetic resonance venography (MRV), or digital subtraction angiography (DSA), including T1-weighted imaging (T1WI) and T2-weighted imaging (T2WI) sequences MRI sequences to analyze thrombosis-related imaging information. The exclusion criteria were: (1) insufficient patient information, including missing any of the following: laboratory indicators (routine blood tests and coagulation tests), imaging information, and assessment of CVST severity (modified Rankin Scale [mRS] and National Institute of Health Stroke Scale [NIHSS]) at admission; (2) history of seizures and antiepileptic drug use; and (3) other interfering intracranial lesions, such as traumatic brain injury, intracranial tumor, brain abscess, or meningitis. This study was conducted in accordance with the ethical guidelines of the 1964 Declaration of Helsinki. This study was approved by the local Ethics Committee (Fujian, China) of the two centers and was exempt from the requirement for informed consent due to its retrospective design.

### Demographic Data and Clinical Variables

Demographic data were collected, including smoking history and chronic diseases (hypertension, diabetes mellitus, dyslipidemia, and abnormal liver function). Whether and when seizures occurred were recorded. Seizures occurring before admission or within 2 weeks after CVST diagnosis were classified as early onset seizures (6). Seizures that occurred before admission were recorded based on witness descriptions. NIHSS and mRS scores at admission were recorded to assess stroke severity in patients with CVST (12, 13). Laboratory data were available for each patient at admission as part of an emergency or routine admission checkup.

### Imaging Evaluation

**Figure 1:**
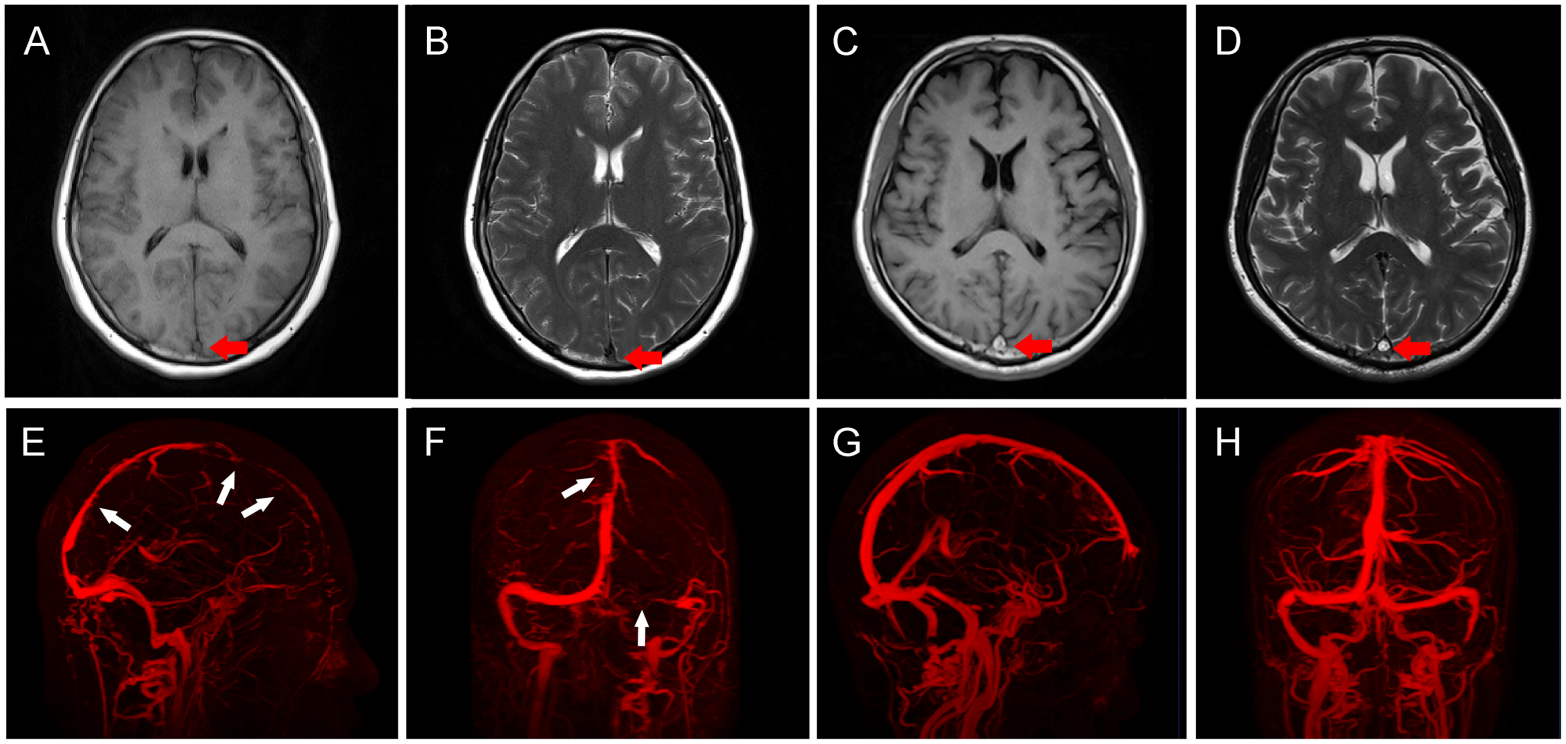
Imaging characteristics of patients with cerebral venous sinus thrombosis (CVST). **(A,B)** In acute CVST patients, clots showed near-normal signal (red arrow) on T1WI (A), and unusual frank hypointensity (red arrow) on T2WI **(B)**. **(C,D)** In subacute CVST patients, clots showed clear hyperintensity (red arrows) on both T1WI **(C)** and T2WI **(D)**. **(E,F)** Three-dimensional reconstruction of MRV in CVST patients. The sagittal view **(E)** showed multiple filling defects in SSS (white arrows), coronal view **(F)** showed filling defects (white arrow) in SSS, and the right transverse sinus was not visualized (white arrow). **(G,H)** Three-dimensional reconstruction of MRV in normal subjects. The sagittal **(G)** and coronal **(H)** views show continuous vessels without filling defects.

Patients were scanned using a 3.0-T MRI scanner (Tim Trio; Siemens Medical Solutions, Erlangen, Germany). CVST can be divided into acute, subacute, and chronic phases according to the course of the disease. It is generally believed that the acute phase occurs within 2 days after onset, the subacute phase is 3 days to 1 month, and the chronic phase is after 1 month (14, 15). These phases can also be reflected in imaging examinations. In the acute phase, clots showed a near-normal signal on T1WI and unusual flank hypointensity on T2WI (Figures 1A,B). In the subacute phase, clots showed clear hyperintensity on both T1WI and T2WI (Figures 1C,D) (16). The distribution of thrombosis was determined using MRV or DSA (Figures 1E,F), compared to MRV in healthy adults (Figures 1G,H) and the corresponding CVST scores were determined according to a previous study (17). Cerebral hemorrhage included intracerebral hematoma and hemorrhagic infarct, and the diagnostic criteria were as reported previously (18). The imaging results were independently evaluated by two neurosurgeons with more than 10 years of experience. If two researchers disagreed, a third researcher (S.W.) made the final decision.

### Statistical Analysis

In this study, statistical analysis was performed using SPSS 18.0 (SPSS Inc, Chicago, IL, USA) and R software (version 4.1.1). Continuous variables are expressed as mean ± standard deviation (SD) or median (interquartile range) and were analyzed using the independent samples *t*-test or Wilcoxon rank-sum test. Categorical variables are expressed as counts (percentages) and were analyzed using Pearson's χ^2^ test or Fisher's exact test. Independent risk factors for seizures in patients with CVST were assessed using a logistic regression analysis. All variables in the univariate analysis (P <0.05) were included in the multivariate regression analysis, and backward stepwise regression was performed to create the final model. The least significant variables in each analysis were sequentially removed once a time from the model until *P* < 0.05 for the remaining variables. Receiver operating characteristic (ROC) curves were used to calculate the area under the curve (AUC), and the maximum value of the Youden index (sensitivity + specificity – 1) was calculated to obtain the best cut-off points. Statistical significance was set at *P* < 0.05.

## Results

### Patient Characteristics

A total of 112 CVST patients (63 men and 49 women; mean age 39.82 ± 15.70 years) were included in this study (Table 1). Thirty-four (30.36%) patients developed early onset seizure and 78 (69.64%) patients did not. The demographics, medical history, clinical characteristics, and laboratory tests were compared between the two groups, and the results are shown in Table 1.

**Table 1:** Demographic features and clinical characteristics of CVST patients with and without early-onset seizures.

**Characteristics**	**Total** **(*n* = 112)**	**Seizure** **(*n* = 34)**	**No seizure** **(*n* = 78)**	***P*** **value**
**Demographics**				
Age, yrs	39.82 ± 15.70	35.38 ± 12.21	41.76 ± 16.70	0.077
Gender, female	49 (43.75%)	15 (44.12%)	34 (43.59%)	0.959
**Medical history**				
Smoking history	23 (20.54%)	8 (23.53%)	15 (19.23%)	0.605
Hypertension	21 (18.75%)	5 (14.71%)	16 (20.51%)	0.469
Diabetes mellitus	16 (14.29%)	5 (14.71%)	11 (14.10%)	0.933
Dyslipidemia	33 (29.46%)	9 (26.47%)	24 (30.77%)	0.646
Abnormal liver function	19 (16.96%)	4 (11.76%)	15 (19.23%)	0.419
**Admission clinical grade**				
mRS	1 (1–2.75)	1 (1–3)	1 (1–2)	**0.018**
NIHSS	2 (0–8)	6.5 (0–12)	0 (0–4)	**<0.001**
**Imaging indicators**				
cerebral ischemia	42 (37.50%)	15 (44.12%)	27 (34.62%)	0.340
cerebral hemorrhage	56 (50.00%)	27 (79.41%)	29 (37.18%)	**<0.001**
Cortical vein thrombosis	60 (53.57%)	26 (76.47%)	34 (43.59%)	**0.001**
Anterior SSST	21 (18.75%)	14 (41.18%)	7 (8.97%)	**<0.001**
Middle SSST	51 (45.54%)	21 (61.76%)	30 (38.46%)	**0.023**
Posterior SSST	59 (52.68%)	21 (61.76%)	38 (48.72%)	0.204
Intracranial deep vein thrombosis	22 (19.64%)	9 (26.47%)	13 (16.67%)	0.230
CVST score	3 (2–5)	4 (2.75–5)	3 (2–5)	**0.047**
**Laboratory**				
WBC, × 10^9^/L	9.53 ± 4.23	10.60 ± 5.29	9.06 ± 3.62	0.097
NEU, %	71.13 ± 11.92	75.34 ± 11.28	69.30 ± 11.79	**0.013**
RBC, × 10^12^/L	4.52 ± 0.67	4.54 ± 0.61	4.51 ± 0.70	0.265
HGB, g/L	130.79 ± 23.33	131.79 ± 20.23	130.35 ± 24.67	0.992
PLT, × 10^9^/L	243.84 ± 90.72	220.44 ± 73.12	254.04 ± 96.05	0.091
D-dimer, mg/L	2.99 ± 4.51	4.50 ± 5.43	2.33 ± 3.90	**0.001**
PT, s	12.55 ± 3.94	12.16 ± 2.52	12.72 ± 4.42	0.737
PT-INR	1.10 ± 0.30	1.08 ± 0.26	1.10 ± 0.31	0.854
APTT, s	27.88 ± 6.82	26.17 ± 5.62	28.63 ± 7.18	0.117
TT, s	18.16 ± 3.50	18.49 ± 2.83	18.01 ± 3.76	0.240
FIB, g/L	3.39 ± 1.48	3.32 ± 1.51	3.42 ± 1.48	0.862
GHB, %	5.76 ± 1.29	5.70 ± 1.12	5.79 ± 1.37	0.651

*mRS, modified Rankin Scale; NIHSS, National Institute of Health Stroke Scale; SSST, superior sagittal sinus thrombosis; CVST, cerebral venous sinus thrombosis; WBC, white blood cell; NEU, neutrophil; RBC, red blood cell; HGB, hemoglobin; PLT, platelet; PT, prothrombin time; APTT, activated partial thromboplastin time; TT, Thrombin time; FIB, fibrinogen; GHB, glycated hemoglobin. Bold value refers to routine laboratory tests on admission, mainly including routine blood and coagulation tests*.

### Risk Factors for Early Onset Seizures in CVST Patients

Early onset seizures were more frequent in patients with the following factors: cerebral hemorrhage (*P* < 0.001), cortical vein thrombosis (*P* = 0.001), anterior SSS thrombosis (SSST) (*P* < 0.001), middle SSST (*P* = 0.023), CVST score ≥4 (*P* = 0.001), mRS ≥2 (*P* = 0.029), NIHSS ≥5 (*P* < 0.001), neutrophil (NEU) percentage ≥76.34% (*P* = 0.001), and D-dimer ≥0.88 mg/L (*P* < 0.001). However, no statistical differences were found with respect to age, sex, medical history, liver function, or intracranial deep vein thrombosis between the two groups (Table 2).

**Table 2:** Univariate analysis of risks of early-onset seizures.

**Predictors**	**Univariate analysis**			
	**Seizure (*n* = 34)**	**No Seizure (*n* = 78)**	**OR (95%CI)**	***P*** **value**
Cerebral hemorrhage	27 (79.41%)	29 (37.18%)	6.52 (2.52–16.85)	<0.001
Cortical vein thrombosis	26 (76.47%)	34 (43.59%)	4.21 (1.69–10.45)	0.001
Anterior SSST	14 (41.18%)	7 (8.97%)	7.1 (2.52–19.97)	<0.001
Middle SSST	21 (61.76%)	30 (38.46%)	2.59 (1.13–5.92)	0.023
CVST score ≥ 4	23 (67.65%)	27 (34.62%)	3.95 (1.68–9.30)	0.001
mRS ≥ 2	22 (64.71%)	33 (42.31%)	2.50 (1.09–5.76)	0.029
NIHSS ≥ 5	19 (55.88%)	15 (19.23%)	5.32 (2.21–12.83)	<0.001
NEU% ≥ 76.34%	21 (61.76%)	23 (29.49%)	3.86 (1.66–9.00)	0.001
D-dimer ≥ 0.88 mg/L	30 (88.24%)	39 (50.00%)	7.50 (2.41–23.31)	<0.001

*The cut-off points of factors were calculated on the basis of ROC curve analysis. SSST, superior sagittal sinus thrombosis; CVST, cerebral venous sinus thrombosis; mRS, modified Rankin Scale; NIHSS, National Institute of Health Stroke Scale; NEU, neutrophil*.

### Predictive Factors of Early Onset Seizure in CVST Patients

**Figure 2:**
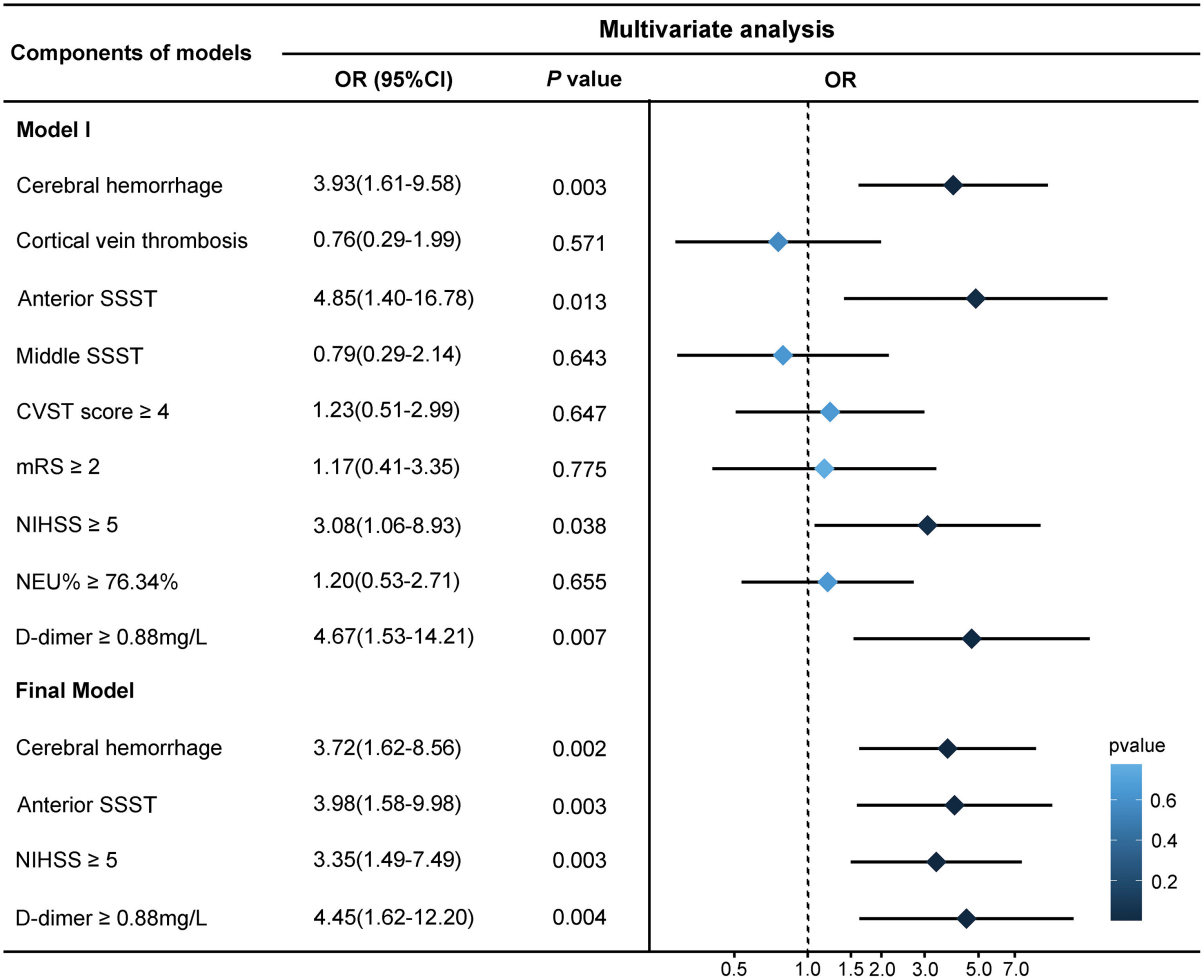
Logistic regression analysis of risk factors for early onset seizures. The cut-off points for factors were calculated on the basis of receiver operating characteristic (ROC) curve analysis. The backward stepwise regression approach was performed to create the final model, in which the least non-significant variable was removed one at a time from the model until all remaining variables were *P* < 0.05. The forest plot on the right demonstrates the results. SSST, superior sagittal sinus thrombosis; CVST, cerebral venous sinus thrombosis; mRS, modified Rankin Scale; NIHSS, National Institute of Health Stroke Scale; NEU, neutrophil.

Variables that may be associated with seizures were analyzed using univariate analysis, and variables with *P* < 0.05 were included in the multivariate analysis (Figure 2). Logistic regression analysis showed that cerebral hemorrhage (odds ratio [OR] = 3.72, 95% confidence interval [CI] = 1.62–8.56, *P* = 0.002), anterior SSST (OR = 3.98, 95% CI = 1.58–9.98, *P* = 0.003), NIHSS ≥5 (OR = 3.35, 95% CI = 1.49–7.49, *P* = 0.003), and D-dimer ≥0.88 mg/L (OR = 4.45, 95% CI = 1.62–12.20, *P* = 0.004) were all significant predictive factors of early onset seizure in CVST patients.

**Figure 3:**
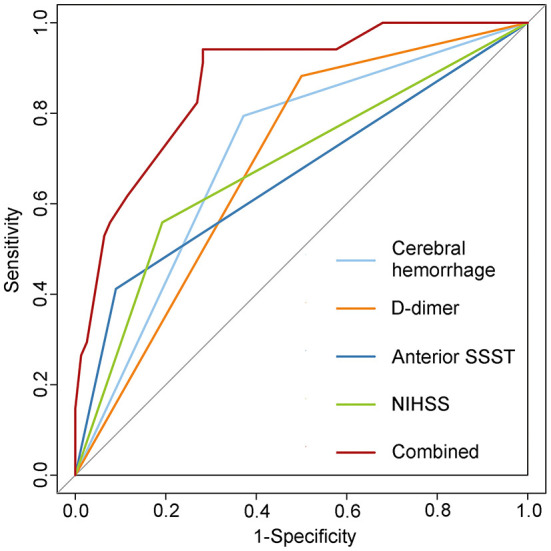
Receiver operating characteristic (ROC) curve of the predictive model for early onset seizures. Combining four predictive factors further improved the predictive capacity as seen in the logistic regression model. SSST, superior sagittal sinus thrombosis; NIHSS, National Institute of Health Stroke Scale.

The AUC of the ROC curve for the predictive performance of cerebral hemorrhage was 0.711 (95% CI = 0.609–0.813). The AUC for the anterior SSST was 0.661 (95% CI = 0.543–0.779). The AUC for D-dimer was 0.691 (95% CI = 0.591–0.792), and a D-dimer level of 0.88 mg/L was the optimal threshold, with a sensitivity and specificity of 88.24 and 50.00%, respectively. The AUC of NIHSS ≥5 was 0.683 (95% CI = 0.570–0.796), and NIHSS ≥5 was the optimal threshold, with a sensitivity and specificity of 61.80 and 76.90%, respectively. When the four predictive factors were combined, the resulting AUC of the ROC curve was 0.871 (95% CI = 0.803–0.939) (Figure 3).

## Discussion

In this study, we evaluated the risk factors associated with early onset seizures in patients with CVST. In the seizure group, cerebral hemorrhage, stroke severity (mRS, NIHSS), thrombosis distribution (cortical vein thrombosis, anterior SSST, CVST score), and laboratory tests (NEU%, D-dimer) were more severe than in those without seizures. After adjusting for confounding factors, cerebral hemorrhage, anterior SSST, NIHSS ≥5, and D-dimer ≥0.88 mg/L were significant independent risk factors.

In both arterial and venous strokes, seizures are common serious complications and are often associated with a more severe prognosis (5, 19). Previous studies have found that anterior cerebral circulation infarction is significantly associated with seizures (20, 21). In contrast, seizures often occur in the frontal lobe, which is supplied by the anterior circulation (22). In this study, we found that anterior and middle SSSTs were risk factors for seizures, with anterior SSST being an independent risk factor. It has been reported that SSS mainly collects cortical venous blood from the frontal, parietal, and superior occipital lobes (23). In addition, Zhang et al. proposed the concept of a “vascular neural network,” showing that the normal physiological function of brain needs to be maintained by both stable arterial and venous blood flow (24). Therefore, it can be supposed that anterior SSST may partially affect stable physiological activity in the frontal lobe by further blocking venous return. In a previous study, Uluduz et al. found that SSST was an independent risk factor for seizures in patients with CVST, although there was no subgroup analysis of the specific location of thrombosis in the SSS (8). As cerebrospinal fluid flows into the SSS via arachnoid granulations, specific structures present inside the SSS may seriously affect cerebrospinal fluid absorption and increase intracranial pressure, potentially inducing the onset of seizures (25). This mechanism may be more pronounced in acute phase patients. Thus, for patients with anterior SSST, the restriction of CSF return may be more severe, which may be another reason for their greater susceptibility to seizures. Interestingly, cortical vein thrombosis was not found to be an independent risk factor for seizures in this study, which differs from previous findings (5, 9). However, univariate analysis results suggest that cortical vein thrombosis was still a risk factor (*P* = 0.001, OR = 4.21). One possible explanation is that anteromedial SSST, as a confluent access route to the cortical veins, may have masked the predictive role of cortical vein thrombosis. Thrombosis within the SSS is more easily detected than that in cortical veins using MRV or DSA in clinical practice.

In this study, we found that CVST with hemorrhagic and not ischemic stroke may be a predictive factor for seizures. Similarly, a retrospective study by Mahale et al. showed that cerebral hemorrhage was the most significant risk factor for early onset seizures (10). Additionally, in a prospective multicenter study of 194 patients with acute CVST, Masuhr et al. found that the risk of seizures after cerebral hemorrhage was 2.8 times higher than that in patients without cerebral hemorrhage (5). In addition, a recent system-review showed that cerebral hemorrhage is a negative factor affecting outcome of patients with intracranial deep vein thrombosis (26). Similar results have been reported for arterial stroke (27). Thus, the blood may be a potential factor that leads to seizures. In this process, extravasated and lysed erythrocytes release free iron, an oxidant that may induce the production of free radicals, reactive oxygen species, and damage oxidant-sensitive cellular enzymes, thereby causing injury to the central nervous system (28). Using rat models, Gong et al. observed that brain injury was effectively alleviated by inhibiting erythrocyte lysis and iron deposition after cerebral hemorrhage (29). Iron reagents have been used in animal studies for the construction of seizure models owing to their epileptic effects (30, 31). Unlike cerebral hemorrhage after arterial stroke, cerebral hemorrhage in CVST often shows a scattered distribution, which may be related to different hemodynamic characteristics of arterial and venous system function. The function of the arterial system depends mainly on the pumping force of the heart and elasticity of the vessels. In contrast, the function of the venous system is related to the venous pressure difference; the venous pressure gradually decreases in cortical veins until the SSS pressure drops further, causing blood flow. Therefore, venous pressure of SSS exceeds the cortical veins reversely after SSST, which may lead to the rupture of cortical veins, and in turn manifests as scattered cerebral hemorrhage (23). This scattered hemorrhage may also be a potential mechanism why patients with CVST are more prone to seizures, and the scattered distribution of hematomas increases the contact area between blood and brain tissue.

This study found that NIHSS score was different between the “seizure” and “no seizure” groups, suggesting that patients with severe stroke are more likely to develop seizure. The NIHSS is a scale used to assess stroke severity that provides a quantitative measure of key components of standard neurological examinations, and its reliability and validity have been demonstrated in retrospective and prospective clinical studies (12). While the mRS was also used to assess the severity of stroke, there was no significant difference in the multivariate analysis model in this study. One possible reason is that the NIHSS is more sensitive than the mRS as a direct measure of cognition, language, visual function, and motor function, particularly in analyses with small sample sizes (32). D-dimer ≥0.88 mg/L was also a predictive factor of seizure in this study. D-dimer level is a marker of endogenous fibrinolysis and is expected to correlate with the degree of venous thrombosis. Misra et al. found that D-dimer levels were helpful in the diagnosis of CVST (33). However, D-dimer is characterized by high sensitivity and low specificity, generally between 40 and 60%, and may present false positives in cases of acute inflammation, surgery or trauma, and tumors (34). A multicenter prospective study on the diagnosis of CVST by Heldner et al. showed that risk prediction for patients who may develop CVST was achieved by using a new clinical score combined with D-dimer levels (35). Similarly, when combining cerebral hemorrhage, anterior SSST, NIHSS, and D-dimer, the AUC of the predictive model was 0.871 (95% CI = 0.803–0.939). This suggests that the combined consideration of these factors results in a greater improvement in the predictive effect of seizures.

This study inevitably had limitations, mainly because of its retrospective study design. Similar to previous CVST clinical studies, this study has the inherent limitation of relatively insufficient data, which limits statistical analysis. Therefore, the research results should be interpreted cautiously. Further large-scale prospective studies are required to confirm these findings.

## Conclusion

In summary, the stroke severity, imaging results, and laboratory tests available during admission evaluation may contribute to the identification of CVST patients at high risk of early onset seizures. Among them, cerebral hemorrhage, anterior SSST, NIHSS, and D-dimer are valuable predictive factors of concurrent seizure in patients with CVST, while combining these four factors further improves predictive capability.

## Data Availability Statement

The raw data supporting the conclusions of this article will be made available by the authors, without undue reservation.

## Ethics Statement

The studies involving human participants were reviewed and approved by Fuzong Clinical Medical College and Affiliated Fuzhou First Hospital of Fujian Medical University. Written informed consent for participation was not required for this study in accordance with the national legislation and the institutional requirements.

## Author Contributions

SM: conceptualization, formal analysis, methodology, and writing—original draft. JL: investigation, methodology, and writing—original draft. KL: formal analysis and data curation. YF: formal analysis and software. FL and ZL: data curation. YX: funding acquisition, visualization, and supervision. SW: funding acquisition, project administration, supervision, validation, and writing—review & editing. All authors contributed to the article and approved the submitted version.

## Funding

This work was supported by Startup Fund for scientific research at Fujian Medical University (2020QH2040); Fujian Provincial Science and Technology Innovation Joint Fund (2019Y9045); Fujian Provincial Natural Science Foundation of China (2018J01350); China Postdoctoral Science Foundation (2021M693955).

## Conflict of Interest

The authors declare that the research was conducted in the absence of any commercial or financial relationships that could be construed as a potential conflict of interest.

## Publisher's Note

All claims expressed in this article are solely those of the authors and do not necessarily represent those of their affiliated organizations, or those of the publisher, the editors and the reviewers. Any product that may be evaluated in this article, or claim that may be made by its manufacturer, is not guaranteed or endorsed by the publisher.

## Abbreviations

AUC: area under curve
CI: confidence interval
CVST: cerebral venous sinus thrombosis
DSA: digital subtraction angiography
MRI: magnetic resonance imaging
mRS: modified Rankin Scale
MRV: magnetic resonance venography
NEU: neutrophil
NIHSS: National Institute of Health Stroke Scale
OR: odds ratio
ROC: receiver-operating characteristic
SSS: superior sagittal sinus
SSST: superior sagittal sinus thrombosis
T1WI: T1-weighted imaging
T2WI: T2-weighted imaging.
